# Risk factor analysis and establishment of a nomogram model to predict blood loss during total knee arthroplasty

**DOI:** 10.1186/s12891-024-07570-3

**Published:** 2024-06-10

**Authors:** Yikai Liu, Jiangshan Ai, Xue Teng, Zhenchao Huang, Haoshen Wu, Zian Zhang, Wenzhe Wang, Chang Liu, Haining Zhang

**Affiliations:** 1 Department of Joint Surgery, The Affiliated Hospital of Qingdao University, Qingdao, 266000 China; 2 Department of Orthopaedics and Traumatology, Beijing Jishuitan Hospital, Capital Medical University, Beijing, 100035 China; 3 Department of Thoracic Surgery, The Affiliated Hospital of Qingdao University, Qingdao, 266000 China; 4 Department of Operating Room, The Affiliated Hospital of Qingdao University, Qingdao, 266000 China

**Keywords:** Total knee arthroplasty, Blood loss, Risk factor, Prediction model, Nomogram

## Abstract

**Purpose:**

The risk factors for excessive blood loss and transfusion during total knee arthroplasty (TKA) remain unclear. The present study aimed to determine the risk factors for excessive blood loss and establish a predictive model for postoperative blood transfusion.

**Methods:**

This retrospective study included 329 patients received TKA, who were randomly assigned to a training set (*n* = 229) or a test set (*n* = 100). Univariate and multivariate linear regression analyses were used to determine risk factors for excessive blood loss. Univariate and multivariate logistic regression analyses were used to determine risk factors for blood transfusion. R software was used to establish the prediction model. The accuracy and stability of the models were evaluated using calibration curves, consistency indices, and receiver operating characteristic (ROC) curve analysis.

**Results:**

Risk factors for excessive blood loss included timing of using a tourniquet, the use of drainage, preoperative ESR, fibrinogen, HCT, ALB, and free fatty acid levels. Predictors in the nomogram included timing of using a tourniquet, the use of drainage, the use of TXA, preoperative ESR, HCT, and albumin levels. The area under the ROC curve was 0.855 (95% CI, 0.800 to 0.910) for the training set and 0.824 (95% CI, 0.740 to 0.909) for the test set. The consistency index values for the training and test sets were 0.855 and 0.824, respectively.

**Conclusions:**

Risk factors for excessive blood loss during and after TKA were determined, and a satisfactory and reliable nomogram model was designed to predict the risk for postoperative blood transfusion.

**Supplementary Information:**

The online version contains supplementary material available at 10.1186/s12891-024-07570-3.

## Introduction

Total knee arthroplasty (TKA) is a common and reliable procedure for the treatment of end-stage knee osteoarthritis (OA) [1]. Successful TKA not only requires effective relief of joint pain and accurate alignment of the lower extremities but also minimizes tissue damage and blood loss. In previous investigations, the rate of blood transfusion in TKA procedures was 18.2% in a study involving 139,804 patients^2^ and 9.27% in another involving 949 patients [2]. Therefore, blood management is crucial for individuals undergoing TKA, and preventive measures are needed for those at a high risk for excessive blood loss to avoid or, at least mitigate, the adverse effects of this surgical complication.

Blood transfusion(s) itself is associated with several potential complications, including increased length of hospital stay (LOS), surgical site infection, pathogen transmission, hemolytic transfusion reactions, transfusion-induced coagulopathy, immunological reactions, and acute kidney injury [3–5]. These risks and the associated transfusion costs highlight the importance of minimizing the use of blood products in TKA [3]. 

Previous studies have reported that female sex, older age, longer operative duration, and lower preoperative hemoglobin levels are risk factors for perioperative transfusion after total hip arthroplasty (THA) [2, 6, 7]; however, risk factors for blood loss after TKA are not well understood. As such, the aim of the present study was to determine such risk factors for TKA and to establish a prediction model for blood transfusion, which is crucial for early identification and intervention in patients at high risk for excessive blood loss and blood transfusion.

## Materials and methods

### Patients and data collection

Data from patients, who underwent TKA for OA between September 2022 and May 2023 at the authors’ hospital, were enrolled. Patients with a history of coagulation disorders and those who received allogeneic blood transfusions within 30 days before TKA were excluded from the study. A total of 348 patients were enrolled in the study. After excluding 19 patients with incomplete HCT data, 329 patients were included, and all patients were randomly assigned to either the training group (*N* = 249) or the test group (*N* = 100) for further analysis and establishment of a prediction model for transfusion.

### Surgical technique

All the surgeries were performed by the same experienced surgeon, who have performed over 5000 TKAs. The surgical procedures were performed under general anesthesia. All the participants used posterior stabilized knee prosthesis (Zimmer). The timing of using a tourniquet included early inflation (inflation before skin incision and release after wound closure) and late inflation (inflation before the placement of prothesis and release after the closure of joint capsule). Medial parapatellar approach was used. After the femur and tibia osteotomy, soft tissue was released to achieve balance between internal and external gaps and balance between flexion and extension gaps. After placing the prothesis, the incision was closed layer by layer. 1.5 g (15 ml) TXA or 15 ml normal saline was injected into the joint cavity after suturing the joint capsule. For patients with drainage tubes, the tubes were clamped for 2 h before opening, and was removed 24 h postoperatively. No intraoperative complication was observed. All patients followed the same rehabilitation program and analgesia pattern after surgery.

### Potential predictive factors

Data were obtained from the hospital’s electronic information system. Baseline (age, gender, height, weight, body mass index (BMI), and blood pressure), intraoperative (operation time, timing of using a tourniquet, the use of MAKO robot, the use of drainage), and preoperative laboratory investigation results (hemoglobin (HGB) level, red blood cell count (RBC), hematocrit (HCT), platelet count (PLT), erythrocyte sedimentation rate (ESR), plasma albumin (ALB) and globulin, blood glucose, blood uric acid, blood calcium, blood potassium, blood lipids and hemagglutination index) were also obtained. Hematocrit (HCT) levels were also measured on the morning of postoperative day 1.

### Outcome measures

Total blood loss (TBL) was calculated using the following methods. The estimated blood volume (EBV) was calculated according to Gross formula: EBV = K1 × height (m)^3 + K2 × weight (kg) + K3. When the patient is male, K1 = 0.3669, K2 = 0.03219, K3 = 0.6041, and when the patient is female, K1 = 0.3561, K2 = 0.03308, K3 = 0.1833 [8]. Then, TBL was also calculated according to Gross’s formula: TBL = EBV× (preoperative HCT- postoperative HCT)/average value of HCT [9] The TBL/estimated blood volume (EBV) ratio was calculated as an indicator of evaluate the effect of blood loss. Patients with a TBL/EBV ratio > 20% underwent postoperative blood transfusion(s).

### Statistical analysis

Data were analyzed using SPSS (IBM Corporation, Armonk, NY, USA) (Version 25.0; IBM) and R version 4.1.2 (R Foundation for Statistical Computing, Vienna, Austria) (R Foundation for Statistical Computing). Categorical variables were analyzed using the χ [6] test, and continuous variables were analyzed with the independent samples *t*-test or rank sum test. Differences with *P* < 0.05 were considered to be statistically significant.

#### Identification of risk factors for intra-and postoperative excessive blood loss

**Fig. 1:**
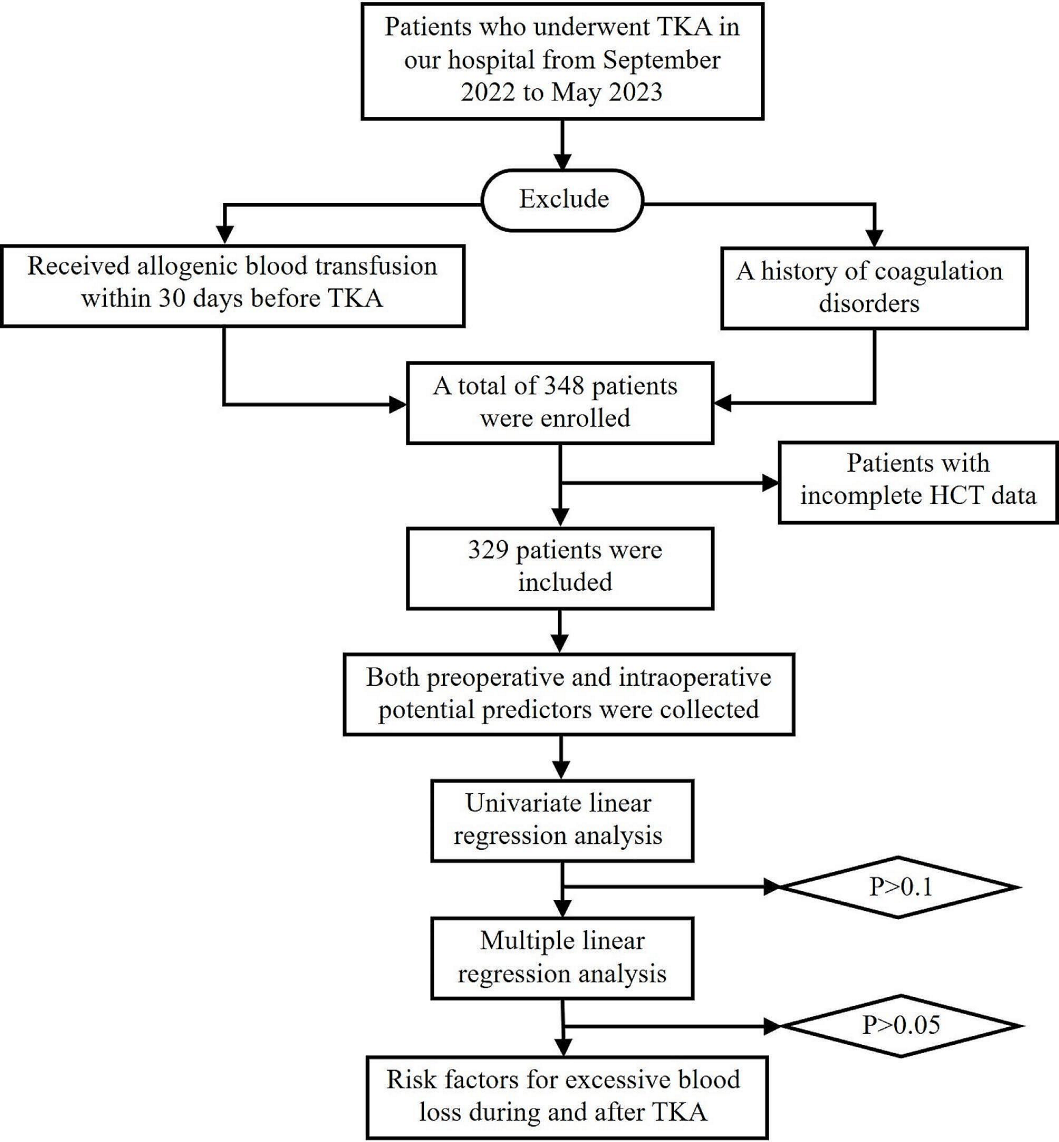
Detailed steps for identifying the risk factors for excessive blood loss during and after TKA

Univariate and multiple linear regression analyses were performed to determine risk factors for excessive blood loss during and after TKA. The dependent variable was EBL/TBV in the univariate and multiple linear regression analyses. Variables were initially screened by univariate linear analysis; those with *P* < 0.1 were included in multivariate linear analysis. Variables with *P* < 0.05 in multivariate linear analysis were regarded as independent risk factors for excessive blood loss during and after TKA. Detailed steps for identifying the risk factors for excessive blood loss during and after TKA are presented in Fig. 1.

#### Establishment of the nomogram prediction model for postoperative blood transfusion

To identify risk factors for postoperative blood transfusion and establish a predictive model. Data from the 329 enrolled patients were randomly divided into training and test sets (*N* = 98). The dependent variable was whether the patients received postoperative blood transfusions in the univariate and multivariate logistic regression analyses. Binary univariate logistic regression analysis was first performed on the potential predictive factors, and those with *P* < 0.1 in the univariate analysis were included in the stepwise backward likelihood ratio (LR) multivariate analysis. Variables with *P* < 0.05 were regarded as independent risk factors for postoperative blood transfusion. Variance inflation factors (VIFs) and tolerances were calculated to assess the collinearity assumption, with a VIF of < 5 and tolerance > 0.1 considered to indicate no significant collinearity. These variables were used to establish a nomogram prediction model.

#### Validation of the nomogram prediction model

**Fig. 2:**
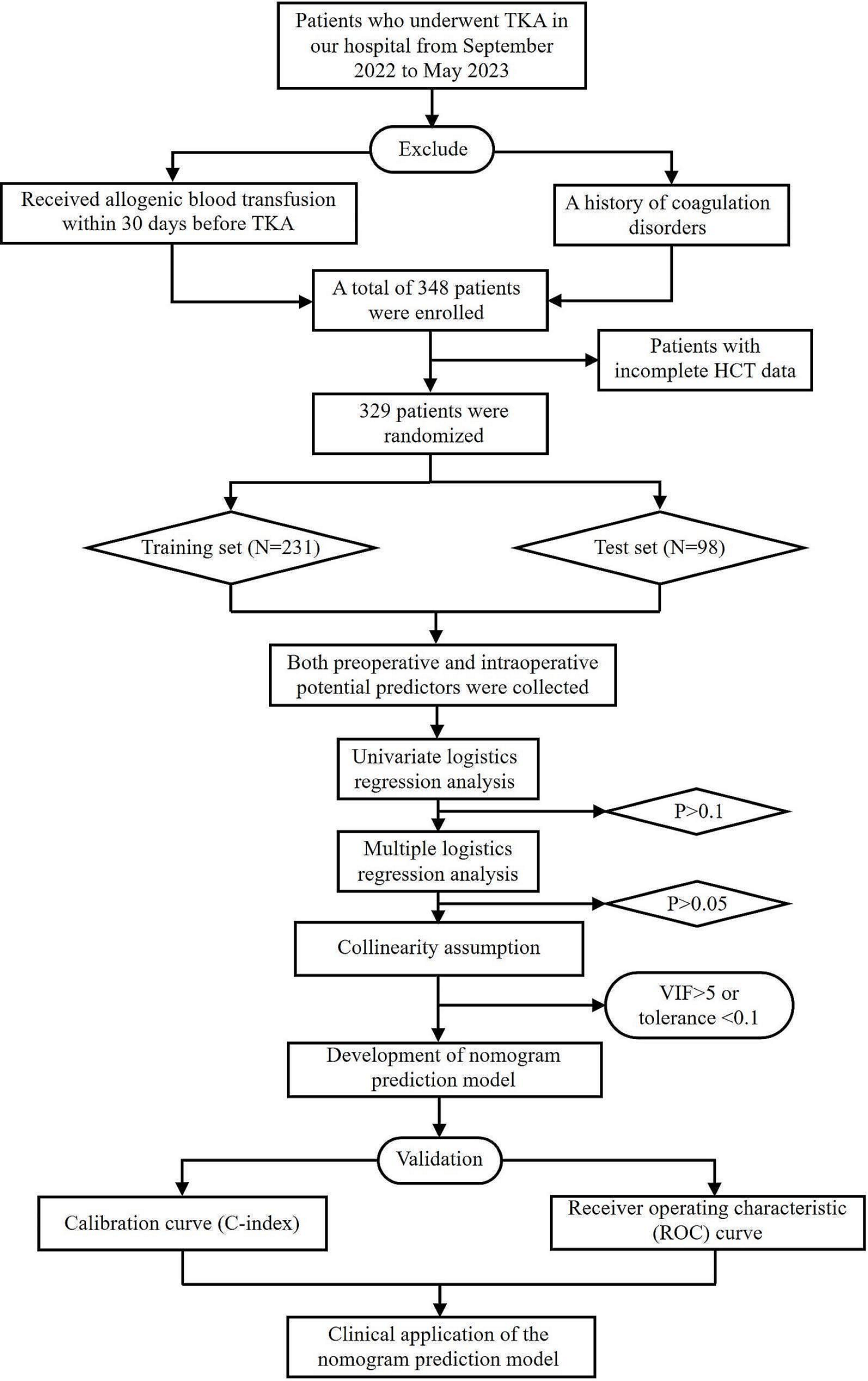
Detailed steps for establishing and validating the nomogram prediction model

The reliability of the internal validation was assessed using the bootstrap method with 1000 replicates. The discrimination of the nomogram model was evaluated using the consistency index (C-index) and receiver operating characteristic (ROC) curve analysis. Calibration curves were drawn, and decision curve analysis (DCA) was performed. The construction and validation of the nomogram prediction model were completed through R language code packages including “rms”, “calibrate”, and “regplot”. The steps for establishing and validating the nomogram prediction model are detailed in Fig. 2.

## Results

### Patient characteristics

A total of 329 patients who fulfilled the inclusion criteria were enrolled in this study. The mean (± SD) age of the cohort was 68.00 ± 6.69 years, and the mean body mass index (BMI) was 27.62 ± 3 0.77 kg/m^2^. Among the 329 patients, 73 received transfusion (mean age, 68.27 ± 6.03 years; mean BMI, 27.27 ± 3.04 kg/m^2^), while 256 did not receive transfusion (mean age, 67.92 ± 6.88 years; mean BMI, 27.80 ± 3.56 kg/m^2^). Other descriptive data for all patients are summarized in Table 1.

**Table 1:** Cohort demographics

	Total (*n* = 329)	Transfusion (*n* = 73)	Non-transfusion (*n* = 256)	Statistics(*t*/χ^2^)	*P*
Age (years)	68.00 ± 6.69	68.27 ± 6.03	67.92 ± 6.88	0.396	0.692
Gender (%)				0.172	0.679
Male Female	84 (25.5) 245 (74.5)	20 (27.4) 53 (72.6)	64 (25.0) 192 (75.0)		
Height (m)	1.62 ± 0.07	1.62 ± 0.07	1.62 ± 0.07	0.012	0.990
Weight (kg)	72.70 ± 10.45	71.70 ± 10.12	72.99 ± 10.55	0.934	0.351
BMI (kg/m^2^)	27.68 ± 3.46	27.27 ± 3.04	27.80 ± 3.56	1.167	0.244
Drainage (%)				3.301	0.069
With Without	132 (40.1) 197 (59.9)	36 (49.3) 37 (50.7)	96 (37.5) 160 (62.5)		
Tourniquet (%)				11.427	0.001
Early Late	221 (67.2) 108 (32.8)	61 (83.6) 12 (16.4)	160 (62.5) 96 (37.5)		
Tranexamic acid (%)				13.914	< 0.001
Use Not use	167 (50.8) 162 (49.2)	23 (31.5) 50 (68.5)	144 (56.3) 112 (43.7)		
ESR (mm/h) Blood cacium Operation time (minutes) Blood glucose (mmol/L) Blood uric acid (µmol/L) ALB (g/L) Globulin (g/L) Triglyceride (mmol/L) Total cholesterol (mmol/L) APOA1 (g/L) APOB (g/L) APOB/APOA1 HDL (mmol/L) LDL (mmol/L) Lipoprotein A (mg/L) Free fatty acids (mmol/L) Blood potassium (mmol/L) Systolic pressure (mmHg) Diastolic pressure (mmHg) HCT (%) Prothrombin time (second) PT percentage activity (%) INR APTT (second) APTT ratio Fibrinogen (g/L) TT (second) TT ratio Activity of antithrombin III (%)	14.79 ± 12.01 2.37 ± 0.14 81.92 ± 27.12 5.90 ± 1.43 342.64 ± 95.33 42.27 ± 3.40 28.48 ± 4.17 1.44 ± 0.83 5.37 ± 1.20 1.58 ± 0.67 0.94 ± 0.27 0.62 ± 0.18 1.52 ± 0.32 3.07 ± 0.93 204.90 ± 233.00 0.60 ± 0.23 4.01 ± 0.37 142.47 ± 18.67 79.83 ± 12.12 41.79 ± 3.70 11.73 ± 1.06 101.18 ± 8.88 0.97 ± 0.18 29.65 ± 5.02 1.04 ± 0.09 3.16 ± 0.66 19.06 ± 11.63 1.17 ± 1.71 94.29 ± 21.44	21.19 ± 18.41 2.41 ± 0.09 84.59 ± 21.19 6.15 ± 1.57 349.85 ± 94.51 43.79 ± 3.61 29.55 ± 4.95 1.66 ± 1.08 5.48 ± 1.20 1.56 ± 0.26 0.98 ± 0.34 0.63 ± 0.19 1.53 ± 0.31 3.09 ± 0.92 216.64 ± 251.82 0.67 ± 0.22 4.02 ± 0.38 143.55 ± 16.80 79.63 ± 12.85 42.19 ± 4.02 11.66 ± 0.90 101.53 ± 8.82 1.00 ± 0.36 28.98 ± 3.95 1.03 ± 0.07 3.27 ± 0.78 17.76 ± 0.88 1.05 ± 0.05 100.63 ± 37.90	12.96 ± 8.64 2.37 ± 0.15 81.16 ± 28.57 5.84 ± 1.38 339.78 ± 97.59 41.83 ± 3.21 28.18 ± 3.88 1.37 ± 0.74 5.34 ± 1.20 1.59 ± 0.74 0.93 ± 0.25 0.62 ± 0.18 1.52 ± 0.32 3.06 ± 0.94 201.55 ± 227.76 0.57 ± 0.23 4.00 ± 0.37 142.16 ± 19.19 79.89 ± 11.92 41.59 ± 3.58 11.75 ± 1.11 101.08 ± 8.91 0.96 ± 0.08 29.84 ± 5.28 1.05 ± 0.09 3.13 ± 0.61 19.43 ± 13.15 1.21 ± 1.93 92.48 ± 13.07	5.382 2.270 0.954 1.651 0.732 4.458 2.497 2.674 0.856 0.319 1.410 0.299 0.266 0.278 0.488 3.294 0.301 0.558 0.159 1.837 0.665 0.386 1.683 1.291 2.007 1.664 1.081 0.715 2.894	< 0.001 0.024 0.341 0.100 0.465 < 0.001 0.013 0.008 0.392 0.750 0.160 0.765 0.791 0.782 0.626 0.001 0.763 0.577 0.873 0.067 0.507 0.699 0.093 0.197 0.046 0.097 0.280 0.475 0.004
D-dimer (µg/mL)	395.53 ± 455.84	430.96 ± 755.63	385.43 ± 324.96	0.752	0.452

Abbreviations: BMI: Body mass index; TXA: Tranexamic acid; ESR: Erythrocyte sedimentation rate; ALB: Albumin; APOA1: Apolipoprotein A1; APOB: Apolipoprotein B; HDL: High density lipoprotein; LDL: Low density lipoprotein; HCT: Hematocrit; INR: International normalized ratio; APTT: Activated partial thromboplastin time; TT: Thrombin time

### Incidence and risk factors for excessive blood loss

#### Univariate linear regression analysis

According to univariate linear analysis, there was no significant correlation between excessive blood loss and age, sex, Kellgren-Lawrence (K-L) grading scale, blood pressure, or preoperative laboratory investigation results, including red blood cells and HGB, PLT, CRP, total cholesterol, lipoprotein A1, lipoprotein B, APOB/APOA, high-density lipoprotein, low-density lipoprotein, lipoprotein A, PT, TT, D-dimer levels. However, there were statistically significant correlation of excessive blood loss with BMI, timing of using a tourniquet, the use of drainage, operation time, and preoperative laboratory test results including HCT, blood calcium, ESR, INR, fibrinogen, blood glucose, activity of antithrombin III, ALB, globulin, triglyceride and free fatty acids. (*P* < 0.1). Analysis results of the measurements and count data are summarized in Table 2.

**Table 2:** Univariate and multivariate linear regression analysis

	Univariate analysis				Multivariate analysis			
	B	SEB	β	*P* Value	B	SEB	β	*P* Value
Age (years)	< 0.001	0.001	-0.02	0.784				
Gender	0.018	0.011	0.087	0.113				
Height (m)	0.05	0.067	0.042	0.452				
Weight (kg)	< 0.001	< 0.001	-0.05	0.33				
BMI (kg/m^2^)	-0.002	0.001	-0.1	0.078	-0.002	0.001	-0.09	0.052
Drainage	0.017	0.01	0.097	0.08	0.017	0.009	0.097	0.048
Tourniquet	-0.036	0.01	-0.19	< 0.001	-0.04	0.009	-0.214	< 0.001
Tranexamic acid	-0.012	0.01	-0.07	0.232				
ESR(mm/h)	0.003	< 0.001	0.356	< 0.001	0.004	< 0.001	0.489	< 0.001
Blood cacium	0.114	0.034	0.18	0.001	0.01	0.032	0.016	0.755
Operation time (minutes)	< 0.001	< 0.001	0.1	0.069	< 0.001	< 0.001	0.024	0.6
Blood glucose (mmol/L)	0.009	0.003	0.14	0.011	0.003	0.003	0.051	0.291
Blood uric acid (µmol/L)	< 0.001	< 0.001	0.087	0.115				
ALB (g/L)	0.008	0.001	0.308	< 0.001	0.005	0.002	0.19	0.002
Globulin (g/L)	0.003	0.001	0.152	0.006	-0.001	0.001	-0.066	0.194
Triglyceride (mmol/L)	0.018	0.006	0.171	0.002	-0.002	0.005	-0.015	0.753
Total cholesterol (mmol/L)	0.003	0.004	0.045	0.417				
APOA1 (g/L)	0.008	0.007	0.06	0.274				
APOB (g/L)	0.012	0.018	0.036	0.512				
APOB/APOA1	-0.02	0.027	-0.04	0.454				
HDL (mmol/L)	0.016	0.015	0.057	0.306				
LDL (mmol/L)	-0.001	0.005	-0.02	0.776				
Lipoprotein A (mg/L)	< 0.001	< 0.001	0.016	0.77				
Free fatty acids (mmol/L)	0.105	0.02	0.273	< 0.001	0.053	0.02	0.138	0.007
Blood potassium (mmol/L)	0.021	0.013	0.089	0.106				
Systolic pressure (mmHg)	< 0.001	< 0.001	0.024	0.671				
Diastolic pressure (mmHg)	< 0.001	< 0.001	-0.01	0.838				
HCT (%)	0.005	0.001	0.2	< 0.001	0.006	0.001	0.265	< 0.001
Prothrombin time (second)	-0.004	0.005	-0.05	0.398				
PT percentage activity (%)	< 0.001	0.001	0.042	0.449				
INR	0.047	0.027	0.099	0.074	0.044	0.022	0.091	0.051
APTT (second)	-0.001	0.001	-0.04	0.462				
APTT ratio	-0.055	0.056	-0.06	0.322				
Fibrinogen (g/L)	0.012	0.007	0.092	0.096	-0.017	0.008	-0.125	0.029
TT (second)	< 0.001	< 0.001	-0.01	0.834				
TT ratio	< 0.001	0.003	-0.01	0.931				
Activity of antithrombin III (%)	0.001	< 0.001	0.139	0.011	< 0.001	< 0.001	0.001	0.976
D-dimer (µg/mL)	< 0.001	< 0.001	0.033	0.548				

Abbreviations: BMI: Body mass index; TXA: Tranexamic acid; ESR: Erythrocyte sedimentation rate; ALB: Albumin; APOA1: Apolipoprotein A1; APOB: Apolipoprotein B; HDL: High density lipoprotein; LDL: Low density lipoprotein; HCT: Hematocrit; INR: International normalized ratio; APTT: Activated partial thromboplastin time; TT: Thrombin time

#### Multivariate linear regression analysis

Multivariate linear regression analysis including 15 statistically significant variables as independent variables, and TBL/EBV as the dependent variable revealed that TBL/EBV was associated with timing of using a tourniquet (*P* < 0.001), drainage (*P* = 0.048), preoperative erythrocyte sedimentation rate (ESR) (*P* < 0.001), fibrinogen (*P* = 0.029), HCT (*P* < 0.001), albumin (ALB) (*P* < 0.002), and free fatty acid levels (*P* = 0.007) (Table 2).

### Incidence and risk factors for blood transfusion

#### Univariate logistic regression analysis

Factors with *P* < 0.1 in the univariate analysis were regarded to be significant. According to univariate logistic analysis, 13 variables were identified as significant factors for transfusion, including the use of drainage, timing of using a tourniquet, the use of TXA, and preoperative HCT, ESR, blood calcium, APTT ratio, fibrinogen, activity of antithrombin III, ALB, globulin, triglyceride and free fatty acid levels, whereas other variables did not exhibit significant differences between the transfusion and non-transfusion groups (Table 3).

**Table 3:** Univariate and multivariate logistic regression analysis

	Univariate analysis		Multivariate analysis	
	OR (95% CI)	*P* value	OR (95% CI)	*P* value
Age (years)	1.008 (0.969–1.048)	0.691		
Gender	1.132 (0.629–2.036)	0.679		
Height (m)	0.978 (0.028–34.612)	0.99		
Weight (kg)	0.988 (0.963–1.013)	0.35		
BMI (kg/m^2^)	0.955 (0.885–1.032)	0.244		
Drainage	1.622 (0.960–2.738)	0.071	2.324 (1.088–4.965)	0.029
Tourniquet	0.328 (0.168–0.640)	0.001	0.142 (0.059–0.338)	< 0.001
Tranexamic acid	0.358 (0.206–0.621)	< 0.001	0.175 (0.084–0.362)	< 0.001
ESR(mm/h)	1.054 (1.029–1.080)	< 0.001	1.122 (1.070–1.177)	< 0.001
Blood cacium	7.739 (0.860-69.678)	0.068	0.905 (0.064–12.702)	0.941
Operation time (minutes)	1.004 (0.995–1.013)	0.353		
Blood glucose (mmol/L)	1.148 (0.972–1.356)	0.105		
Blood uric acid (µmol/L)	1.001 (0.988–1.004)	0.464		
ALB (g/L)	1.217 (1.111–1.333)	< 0.001	1.260 (1.091–1.454)	0.002
Globulin (g/L)	1.082 (1.016–1.152)	0.015	0.964 (0.885–1.051)	0.409
Triglyceride (mmol/L)	1.455 (1.088–1.946)	0.011	1.155 (0.750–1.778)	0.514
Total cholesterol (mmol/L)	1.100 (0.885–1.366)	0.391		
APOA1 (g/L)	0.928 (0.586–1.471)	0.751		
APOB (g/L)	1.947 (0.763–4.969)	0.163		
APOB/APOA1	1.246 (0.295–5.272)	0.765		
HDL (mmol/L)	1.117 (0.495–2.519)	0.79		
LDL (mmol/L)	1.041 (0.787–1.376)	0.781		
Lipoprotein A (mg/L)	1.000 (0.999–1.001)	0.625		
Free fatty acids (mmol/L)	6.691 (2.084–21.483)	0.001	2.834 (0.590-13.607)	0.193
Blood potassium (mmol/L)	1.115 (0.550–2.262)	0.763		
Systolic pressure (mmHg)	1.004 (0.990–1.018)	0.576		
Diastolic pressure (mmHg)	0.998 (0.977–1.020)	0.873		
HCT (%)	1.069 (0.995–1.148)	0.068	1.193 (1.066–1.335)	0.002
Prothrombin time (second)	0.918 (0.714–1.181)	0.506		
PT percentage activity (%)	1.006 (0.977–1.036)	0.698		
INR	2.958 (0.505–17.316)	0.229		
APTT (second)	0.965 (0.914–1.019)	0.198		
APTT ratio	0.039 (0.002–0.949)	0.046	0.033(0.001–2.006)	0.103
Fibrinogen (g/L)	1.380 (0.941–2.023)	0.099	0.662(0.352–1.246)	0.201
TT (second)	0.857 (0.643–1.142)	0.293		
TT ratio	0.081 (0.001–10.379)	0.31		
Activity of antithrombin III (%)	1.027 (1.004–1.050)	0.02	1.002 (0.983–1.021)	0.841
D-dimer (µg/mL)	0.465 (1.000-1.001)	1		

Abbreviations: BMI: Body mass index; TXA: Tranexamic acid; ESR: Erythrocyte sedimentation rate; ALB: Albumin; APOA1: Apolipoprotein A1; APOB: Apolipoprotein B; HDL: High density lipoprotein; LDL: Low density lipoprotein; HCT: Hematocrit; INR: International normalized ratio; APTT: Activated partial thromboplastin time; TT: Thrombin time

#### Multivariate logistic regression analysis

Multivariate logistic regression analysis of 13 statistically significant variables revealed that transfusion was associated with timing of using a tourniquet (odds ratio [OR] = 0.142, 95% confidence interval [CI] = 0.059–0.338, *P* < 0.001), drainage, (OR 2.324, 95% CI 1.088–4.965, *P* = 0.029), the use of TXA (OR 0.175, 95% CI 0.084–0.362, *P* < 0.001), preoperative ESR (OR 1.122, 95% CI 1.070–1.177, *P* < 0.001), HCT (OR 1.193, 95% CI 1.066–1.335, *P* = 0.002), and ALB level (OR 1.260, 95% CI 1.091–1.454, *P* = 0.002) (Table 3).

### Development and validation of nomogram for transfusion

**Fig. 3:**
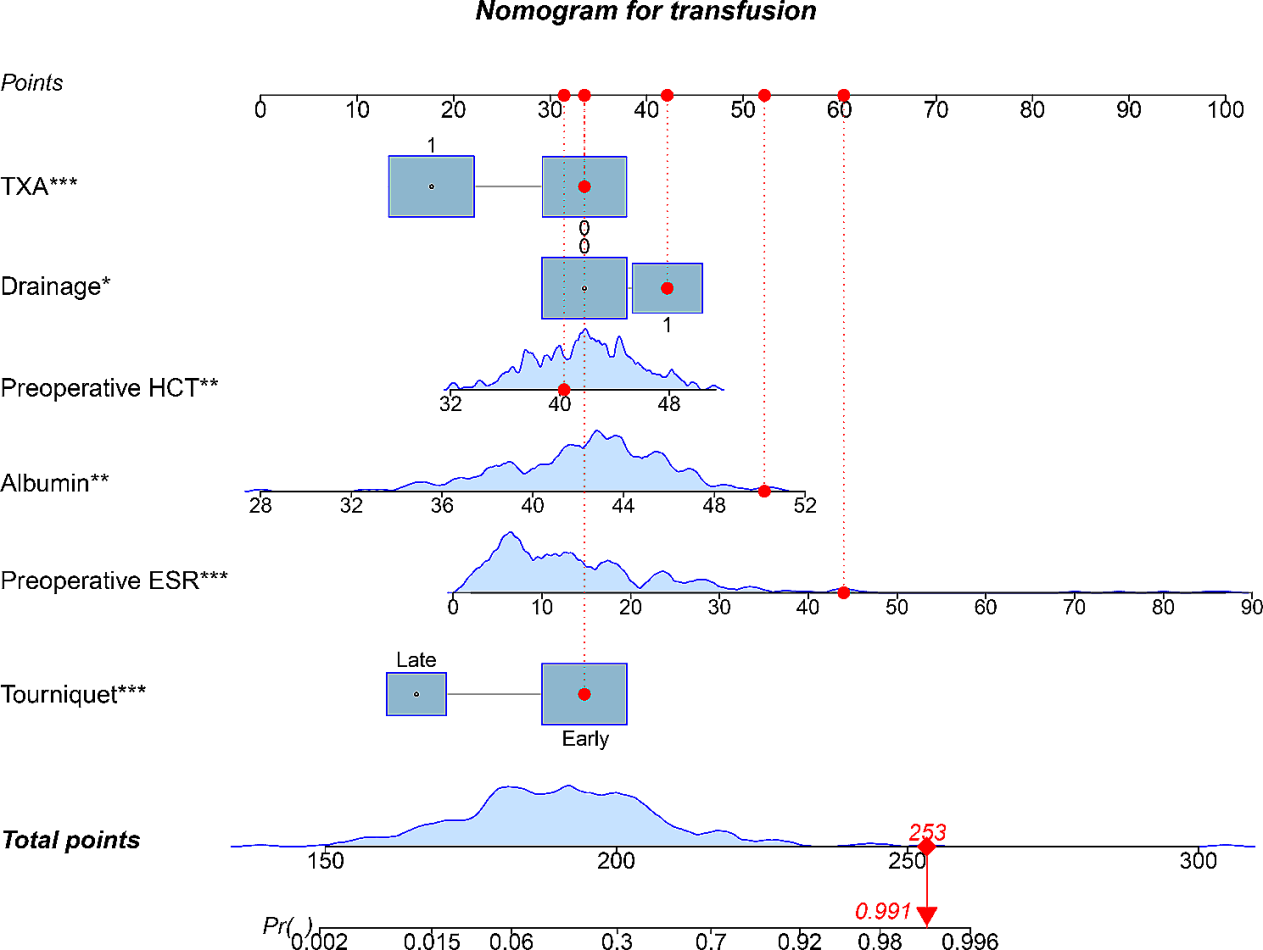
A nomogram based on the 6 independent predictors of transfusion. For the TXA or the Drainage, “0” refers to not use and “1” refers to use; For the Tourniquet, “early” refers to inflation before skin incision and release after wound closure, and “late” refers to inflation before the placement of prothesis and release after the closure of joint capsule. *P < 0.05; **P < 0.01; ***P < 0.001

**Fig. 4:**
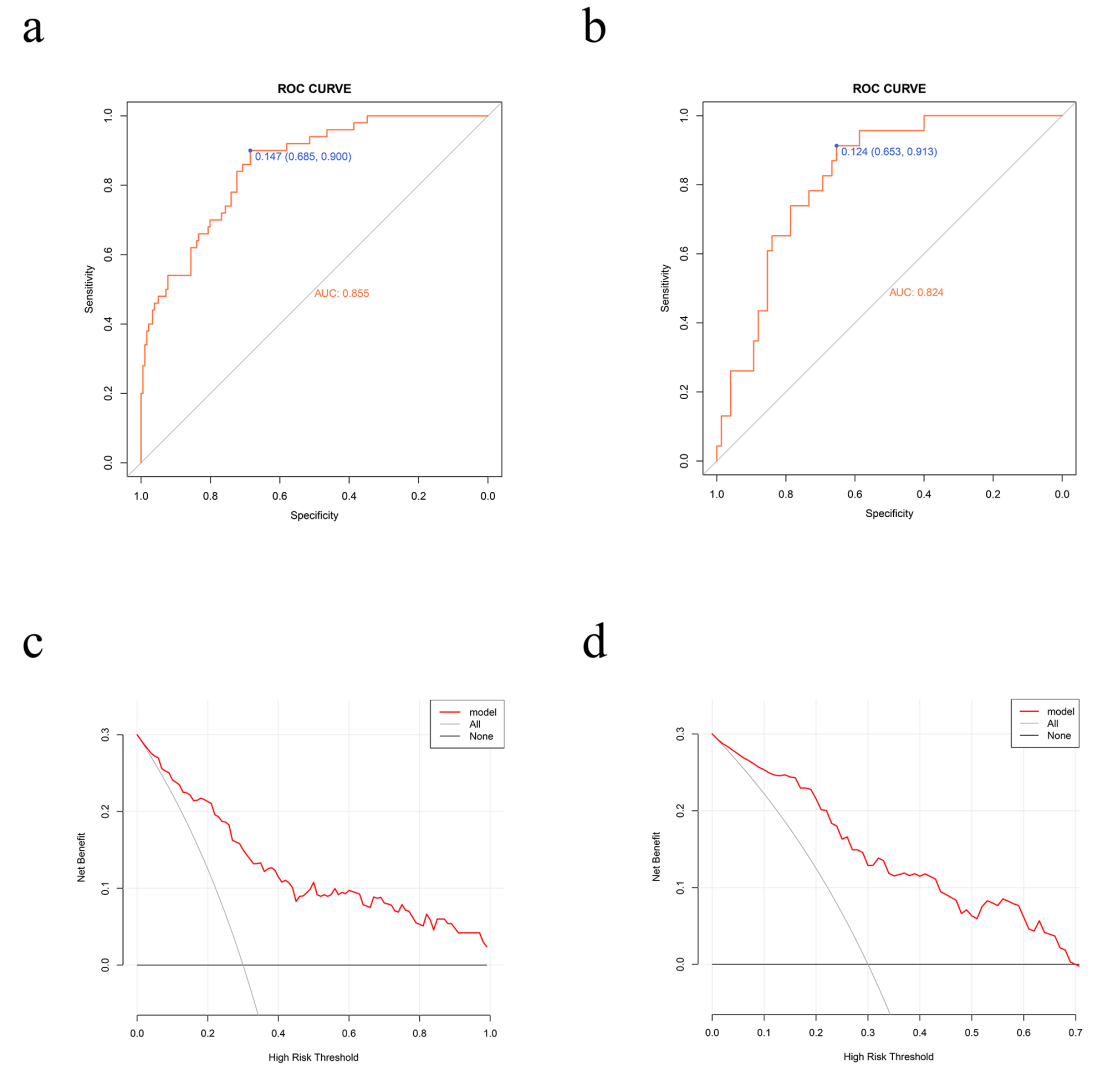
The receiver operating characteristic curve of the training set (**a**) and the test set (**b**). The decision curve analysis of the training set (**c**) and the test set (**d**)

Independent risk factors were included in the logistic regression prediction model and the nomogram was generated. The integral value of each included variable is reported in Fig. 3 and, by combining the integral values of all variables, the total score and corresponding probability can be calculated. The area under the ROC curve (AUC) (Fig. 4) was 0.855 (95% confidence interval [CI] 0.800–0.910) for the training set and 0.824 (95% CI 0.740–0.909) for the test set, indicating satisfactory discrimination performance of the prediction model. Furthermore, the C-index values from the calibration curves were 0.855 for the training set and 0.824 for the test set, and the 95% CI obtained using the bootstrap method with 1000 replicates was 0.800–0.910 for the training model and 0.740–0.909 for the test model. To identify the potential clinical benefits of the designed nomogram, DCA was performed using this dataset. DCA for this model, demonstrating its superiority over “treat all” or “treat none” strategies when threshold probabilities range from 0 to 1 is presented in Fig. 4. The calibration curve demonstrated good agreement with the observed probabilities of blood transfusion in this study (Supplementary file 1). These findings indicate that the prediction model was satisfactory.

## Discussion

Blood loss is inevitable in patients undergoing TKA, and sometimes patients require allogeneic blood transfusion to maintain normal hemoglobin levels. However, several studies have suggested that transfusion increases the risk for complications, mortality, costs, and length of hospital stay [4, 5, 10, 11]. Therefore, it is essential to identify high-risk patients and intervene early to reduce the risk for transfusion. Some studies have investigated single factors that affect blood loss and transfusion. However, few investigations have screened and identified the risk factors for excessive blood loss or established a prediction model to predict the risk for transfusion after TKA.

We propose, for the first time, that a high preoperative HCT value is an independent risk factor for postoperative blood transfusion in TKA. We found that the preoperative HCT in the transfusion group was higher than that in the non-transfusion group. Our study was supported by another study suggesting that a high preoperative HCT value was a risk factor for increased hidden blood loss (HBL) in patients undergoing laparoscopy and laparotomy for cervical cancer treatment [12]. 

The purpose of tourniquet use in TKA is to reduce or―at least mitigate―intraoperative blood loss, provide a better surgical field of vision, and improve the integration of bone cement into the bone. However, some evidence has challenged the necessity and safety of tourniquets because they may induce vascular injury, thromboembolism, reduce range of motion (ROM), and aggravate postoperative pain postoperatively [13–15]. Some researchers argued that no reduction in postoperative transfusion rates is observed when a tourniquet is used. In fact, while the use of tourniquets reduces intraoperative blood loss [16], it significantly increases total blood loss (i.e., blood loss during the operation and in the postoperative period) [17]. Activation of fibrinolysis after tourniquet deflation may explain this phenomenon [18]. Our results indicated that early inflation of tourniquet was an independent risk factor for increased blood loss and postoperative transfusion, which was consistent with the aforementioned findings.

The use of surgical drains after TKA has been standard practice for many years [19]. It is believed that the reduced formation of hematomas may reduce swelling, reduce the nidus for infection, and improve ROM [20]. However, the necessity for drainage in TKA has been a challenged [21]. Some clinicians believe that drainage increases blood loss because it destroys the tamponade effect at the surgical site. Drainage yielded no significant improvement in terms of ROM, reduction in swelling, and LOS [22] and did not increase the risk for complication(s) after TKA [23]. Closed-suction drainage is also a risk factor for infection after TKA [24]. Our results indicated that drainage was an independent risk factor for increased blood loss and postoperative transfusion.

We found that high ALB levels were risk factors for increased blood loss and transfusion. A low ALB level was an independent risk factor for increased LOS in patients undergoing TKA and/or THA. One study investigating TKA suggested that preoperative ALB < 30 g/L could increase LOS [25]. Similar results were found in THA, as the ALB level of patients with LOS < 48 h was significantly higher than that of patients with LOS > 48 h [26]. However, the effect of preoperative ALB levels on blood loss has rarely been reported. One study suggested that ALB was not associated with HBL after posterior lumbar fusion surgery; however, it did not report a correlation between ALB and TBL [27]. Higher preoperative plasma ALB level was an independent risk factor for greater intraoperative blood loss during intracranial meningioma [28]. 

We found that high ESR was an independent risk factor for excessive blood loss and blood transfusion. Our result was supported by another study that proposed that a high ESR was one of the risk factors for blood loss in patients with ankylosing spondylitis with hip involvement undergoing THA [29]. ESR is regarded as a biomarker of inflammatory diseases, and its level reflects disease activity. Although OA has not been traditionally treated as an inflammatory disease, accumulating evidence suggests that inflammation plays a role in its pathogenesis and progression [30–32]. For patients with high preoperative ESR, we believe that systematic history taking is essential to exclude active inflammation. Good management of the preoperative ESR is not only useful for reducing the possibility of infection but also for reducing blood loss during the perioperative period of TKA.

The use of tranexamic acid (TXA) in TKA has been confirmed to be effective in reducing blood loss and the risk for blood transfusion [33]. Our study further confirmed this finding because the transfusion rate in the TXA group was lower than that in the non-TXA group. We did not observe any complications with the use of TXA, and its safety was confirmed in another study, which suggested that intravenous application of TXA within 24 h after TKA led to reduced HBL without an obvious increase in the incidence of VTE or thrombosis [34]. 

Nomograms are widely used for clinical diagnoses and predictions. In the present study, we established a nomogram prediction model for transfusion(s) after TKA. Based on this nomogram, multiple risk factors can be quantified according to transfusion risk. The AUC for the nomogram was 0.855 (95% CI 0.800–0.910), which was higher than the threshold for good performance (AUC > 0.8). Moreover, the nomogram was validated in an independent cohort, and the validation results confirmed that it was effective in predicting the need for perioperative transfusion after TKA.

There are some researches on the risk factors for blood transfusion in TKA. One research reported that preoperative Hb levels and use of TXA was associated with the Hb loss, and preoperative Hb levels was associated with blood transfusion [35]. However, in our study, no significant difference of the preoperative Hb levels between the transfusion group and the non-transfusion group was observed, while the preoperative HCT was associated with transfusion. Another study proposed that posterior cruciate retaining prosthesis and topical use of TXA were preferred to reduce total blood loss [36]. All the participants used posterior stabilized knee prosthesis in our study so we did not evaluate the effect of difference kind of prosthesis on the blood loss and transfusion. What we share in common with these researches are that the use of tourniquet could not reduce blood loss, the use of drainage could increase blood loss and the use of TXA could reduce blood loss. Apart from these, we also determined that high ALB levels and high ESR were independent risk factors for excessive blood loss and blood transfusion in TKA, which has not been reported previously.

However, this study had some limitations, the first of which was that the patient population was derived from a single center and the sample size was relatively small. Although the model demonstrated satisfactory performance on the internal validation set, it lacked an independent external validation set, which could potentially affect its generalizability. Large-scale, multicenter studies should be performed to further enhance and externally validate the model.

## Conclusion

The present study identified risk factors for excessive blood loss and established a predictive model for postoperative transfusion after TKA. Risk factors for excessive blood loss included the timing of tourniquet use, drainage, preoperative ESR, fibrinogen, HCT, ALB, and free fatty acid levels. Predictors in the nomogram included the timing of tourniquet use, drainage, TXA, preoperative ESR, HCT, and ALB level. We established a satisfactory and reliable nomogram prediction model to predict the risk for postoperative blood transfusion and to guide clinical decision-making regarding reducing blood loss. For those patients with more risk factors of blood transfusion and high score on the nomogram, blood preparation preoperatively is recommended.

### Electronic supplementary material

Below is the link to the electronic supplementary material.

Supplementary Material 1

## Data Availability

The data that support the findings of this study are available from the corresponding author upon reasonable request.
