# Supplementary figures and images for: Risk factor analysis and establishment of a nomogram model to predict blood loss during total knee arthroplasty
Source: BMC Musculoskelet Disord. 2024 Jun 10;25:459. doi: 10.1186/s12891-024-07570-3 (PMC11163717; doi:10.1186/s12891-024-07570-3)

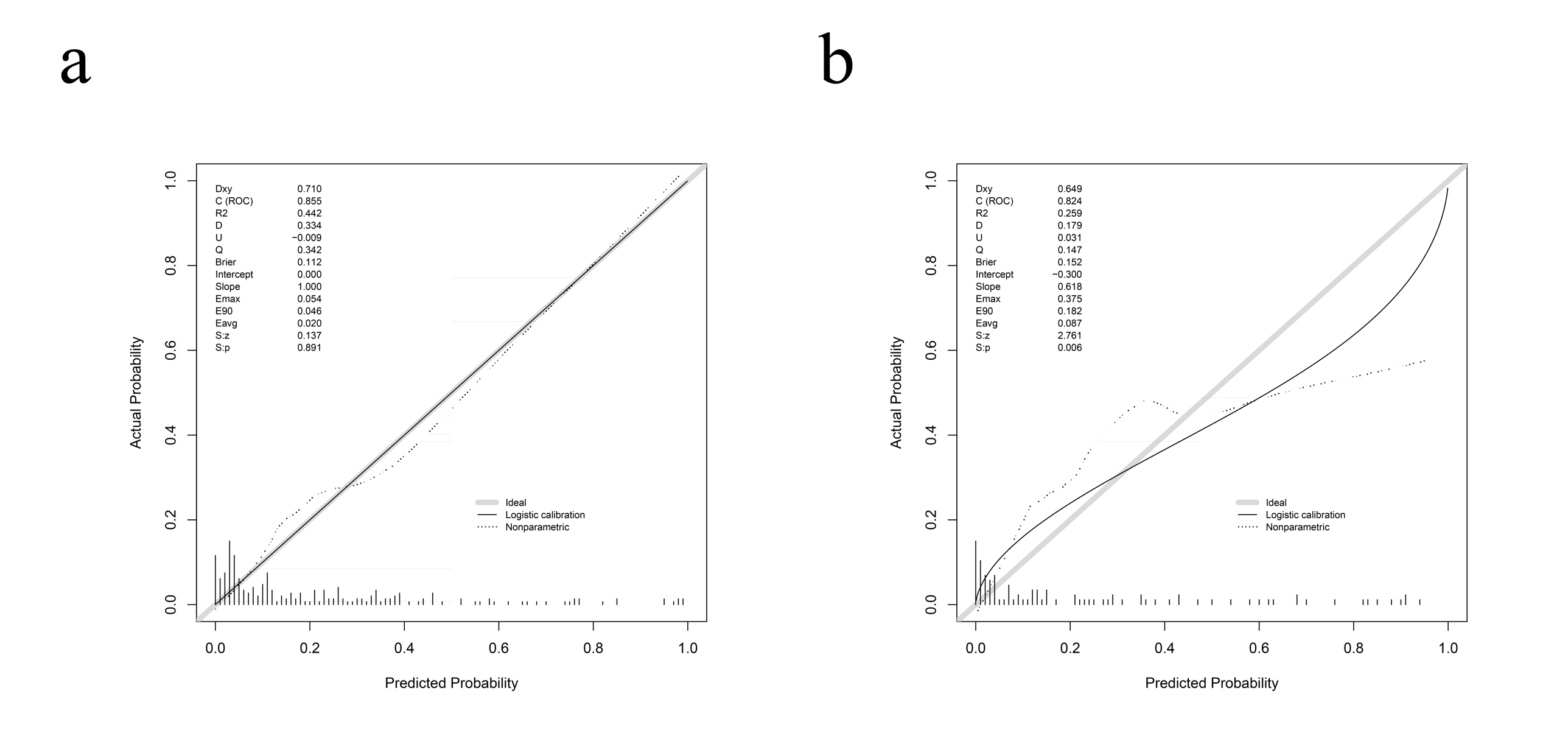

Supplement: Supplementary file 1 — Supplementary Material 1 [file 12891_2024_7570_MOESM1_ESM.png]
